# Review of Organic Photorefractive Materials and Their Use for Updateable 3D Display

**DOI:** 10.3390/ma14195799

**Published:** 2021-10-04

**Authors:** Pierre-Alexandre Blanche, Jae-Won Ka, Nasser Peyghambarian

**Affiliations:** 1 College of Optical Sciences, University of Arizona, Tucson, AZ 85721, USA; nasser@optics.arizona.edu; 2 Advanced Functional Polymers Research Center, Korea Research Institute of Chemical Technology, 141 Gajeong-ro, Yuseong-gu, Daejeon 34114, Korea; jwka@krict.re.kr

**Keywords:** photorefractive, 3D display, holography, energy levels, polymer, electro optic, birefringence, holographic stereogram

## Abstract

Photorefractive materials are capable of reversibly changing their index of refraction upon illumination. That property allows them to dynamically record holograms, which is a key function for developing an updateable holographic 3D display. The transition from inorganic photorefractive crystals to organic polymers meant that large display screens could be made. However, one essential figure of merit that needed to be worked out first was the sensitivity of the material that enables to record bright images in a short amount of time. In this review article, we describe how polymer engineering was able to overcome the problem of the material sensitivity. We highlight the importance of understanding the energy levels of the different species in order to optimize the efficiency and recording speed. We then discuss different photorefractive compounds and the reason for their particular figures of merit. Finally, we consider the technical choices taken to obtain an updateable 3D display using photorefractive polymer. By leveraging the unique properties of this holographic recording material, full color holograms were demonstrated, as well as refreshing rate of 100 hogels/second.

## 1. Introduction

Holograms are known first and foremost for their ability to project 3D images. Holographic 3D images were demonstrated in the early 1960s by Leith, and Upatnieks [1], as well as Denisyuk [2]. What distinguishes holography from other 3D technologies is its ability to reproduce all the human 2D and 3D visual cues: occlusion, parallax, accommodation, and vergence [3,4,5]. This makes holography a strong contender to obtain the ultimate 3D display that would be able to project images as good as the real world [6,7].

From the early work on holographic imaging, many accomplishments have been made regarding the rendering of 3D images using holograms. The depth of field has been enlarged by using long coherence lasers [8]; for example, full color reproduction has been achieved, thanks to 3 color lasers recording [9,10]; reproductions of large scenes and life subjects have been made possible by using short pulse lasers [11]; and rainbow holograms can be viewed with white light [12]. Today, it is possible to display convincing holographic reproductions of artifacts with exquisite details and in full color [13,14].

After the series of successes experienced in the field of static holographic imaging in the first half of the 1960s, there was a good hope that a holographic display presenting motion would appear soon after. Additionally, indeed, the first holographic motion picture was demonstrated by De Bitetto in 1968 and Jacobson in 1969 [15,16]. Holographic motion pictures use a succession of pre-recorded holograms that are projected fast enough to give the impression of movement.

However, motion picture is very different from a refreshable display, such as on a television or computer screen. For these displays, the information could be changed at any moment according to the user input. This requires the display to be dynamic instead of being pre-recorded.

To obtain a dynamic holographic 3D display, several approaches are possible: either using an electronically controlled phase modulator (such as a liquid crystal or acousto-optic modulator) [17,18,19,20], or using a refreshable holographic recording material, such as photorefractive polymer. The present review article focuses on this latter case, specifically the development of photorefractive materials and their use for holographic 3D display.

The organization of the manuscript is as follows: We will first introduce the photorefractive effect in its generality in Section 2. Section 3 is devoted to the chemical engineering of polymer materials to optimize the photorefractive effect. We will discuss the importance of matching the energy levels of the different species, as well as how each type of molecules enhances the effect by its own contribution. Section 4 focuses on the external field and the sample geometry, which both have important consequences for the manufacturing of samples and the use of the material. Finally, Section 5 will review the development of the holographic 3D display in itself and give an overview of the holographic stereographic technique.

## 2. Overview of the Photorefractive Effect

**Figure 1:**
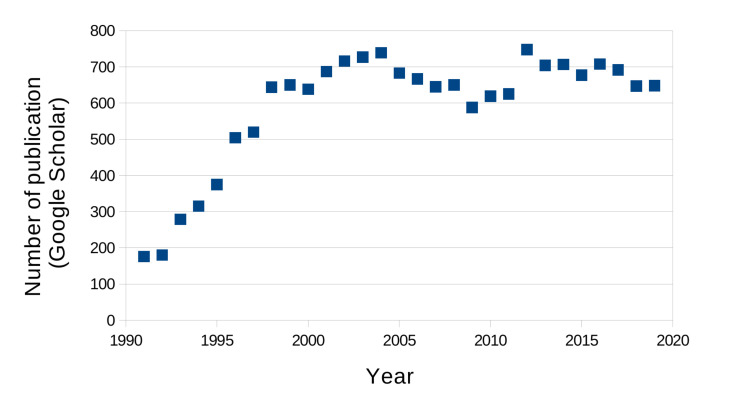
Number of scientific papers mentioning “photorefractive polymer” published over the year, according to Google Scholar.

The photorefractive effect is defined as the reversible change of a material index of refraction upon illumination. Initially observed in inorganic crystals [21], it was then discovered in organic materials [22] and then polymers [23]. Since this original publication, the number of papers mentioning photorefractive polymer has grown over the years to stabilize at around 700 per year. Figure 1 shows this trend obtained from the Google Scholar search website, which indicates that the topic of photorefractive polymers is indeed a very active field of research.

To distinguish the photorefractive effect from other photo-induced index change mechanisms, such as photochromism, photobleaching, or molecular hole burning, the photorefractive effect was specified by its mode of action that involves charge photo-initiation (such as in photovoltaic material), local redistribution of the said charges into a local space-charge field, and, ultimately, the change of the refractive index due to non-linear Pockels effect or molecular reorientation [24].

This seemingly simple effect: a light-induced reversible refractive index change gives rise to a plethora of macroscopic observations and applications, such as self-focusing [25], beam fanning [26], two beam coupling [27], four wave mixing [28], holographic data storage [29], image processing [30], image through scattering media [31], and refreshable holographic recording [32]. For an extended review of the different applications of photorefractive materials, see, for example, References [33,34].

Because the photorefractive effect is based on electronic properties of the material, it is fully reversible, allowing for the recording, the erasing, and the refreshing of holograms at will. To do so, two coherent laser beams are intersecting each other inside the material. One beam is homogeneous and is referred as the reference beam, while the other beam carries the information as an intensity modulation and is referred as the object beam. When these two beams intersect, they form an interference pattern whose frequency is defined by their angle, and its modulation amplitude given by their respective intensities. This interference pattern is copied in the material by the photorefractive effect as a refractive index change.

To retrieve the information stored in the material, a single reading beam is used to illuminate the material. This reading beam is diffracted by the index modulation that was previously recorded, and the resulting diffracted beam reproduces the phase and amplitude that were present in the object beam, i.e., the holographic image.

To erase the hologram, a homogeneous beam is used to illuminate the material, exciting the charges that were trapped in potential wells. These mobile charges recombine, canceling the space-charge electric field. In the absence of space-charge field, the chromophore molecules are free to reorient themselves due to thermal agitation, and the index of refraction returns to an average value. The material is now ready to record a new hologram, eventually different from the first one. This process can be repeated indefinitely since the material does not endure any fatigue during charge generation or molecular orientation.

Compared to other holographic recording materials, such as dichromated gelatin or silver halide, that need post-processing for the hologram to appear, or to photopolymers that record permanent holograms, photorefractive materials represent an interesting platform for the development of an updateable 3D display. However, to do so requires several specific characteristics:

- the material should be sensitive enough so that a reasonable amount of power can be used to write the hologram [35,36],
- the material should be efficient so that high contrast hologram can be recorded [37,38],
- the material should be highly dynamic so thata fast refresh rate can be achieved [39,40],
- the material should be transparent to red, green, and blue light so that color holograms can be displayed [41,42,43],
- the material should withstand a high electric field without breaking down [44,45],
- the material should be processed in large film to achieve large screens that display large holograms [46,47], and
- the material should have a long phase stability so that the display can be used for an extended period of time [48,49].

Combining all these characteristics into a single material is a feat of chemical engineering that had to be developed over many years. In the following section, we retrace the most important discoveries that led to the development of a photorefractive polymer with suitable properties so that it can be used in an updateable 3D display.

## 3. Photorefractive Effect in Polymers

### 3.1. Electronic Behavior in Molecules

Polymer materials can demonstrate photorefractive behavior, thanks to a cascade of events that is important to understand clearly in order to optimize the different figures of merits. Different species of molecules are responsible for each of these events. In the following list, we introduce the different phenomena that led to the photorefractive effect, in their order of appearance, together with the molecular species responsible for it to happen:

1. Charge photo-generation ↔ sensitizer molecules.
2. Charge transport ↔ polymer chains.
3. Charge trapping ↔ conformational traps.
4. Space-charge field ↔ electron and hole differential relocation.
5. Index modulation ↔ chromophore molecules.

The photorefractive process starts by the absorption of a photon by the material and the generation of a bound charge pair. In polymers, to favor the creation of free charges from the bound pair, an external electric field is applied to the material [50,51]. This field helps the dissociation of the charges species: electron and hole, by tilting the potential well. Without an external field, most of the bound pairs recombine and the material returns to a neutral state. To increase the chance of charge creation, strong absorption sensitizer molecules are added to the compound. The sensitizer should have a difference of energy between the HOMO (highest occupied molecular orbital) and LUMO (lowest unoccupied molecular orbital) levels of about 2.5 eV such that it can absorb visible light. Note that, for other spectral bands, such as near-IR [42], the sensitizer can have a shallower band gap. For example, a 2-[2-5-[4-(di-n-butylamino)phenyl]-2,4-pentadienylidene-1,1-dioxido-1-benzothien-3(2H)-ylidene] malononitrile (DBM) sensitizer, in which absorption extends to the near-IR region, has been used to make photorefractive material that operates at 975 nm (= 1.27 eV) [43]. The same sensitizer (DBM) has been used at 1550 nm (= 0.8 eV), thanks to two-photon absorption: 2×0.8=1.6 eV or 775 nm [52,53].

### 3.2. Matching Energy Levels

The sensitizer energy levels should also be compatible with the polymer so that the newly created charge can be transferred to the polymer HOMO manifold. Once there, the charge is free to move by hopping between polymer sites, driven by the external electric field. The charge travels along the polymer chains until it gets fixed into a trap. These traps can be shallow conformational traps, or deep wells created by other molecules, such as the chromophores. Deep traps are usually not favored since they slow down the charge transport and reduce the speed of the photorefractive effect. The dynamic formation of the space-charge field was initially modeled in photorefractive crystals by Moharam et al. [54,55], as well as Kukhtarev et al. [56,57].

**Figure 2:**
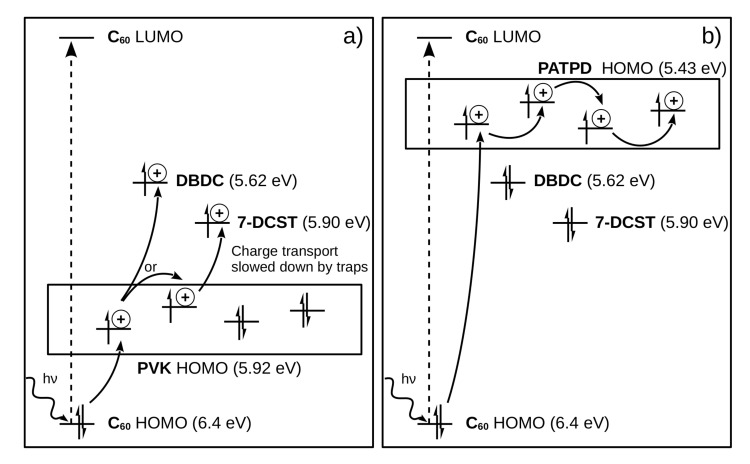
Energy levels of two different photorefractive polymers. (**a**) The chromophores DBDC and 7-DCST can play the role of deep traps for the hole in the PVK polymer matrix, making the PVK photorefractive response slower (>100 ms). (**b**) The PATPD polymer matrix has its HOMO level above the chromophores DBDC and 7-DCST, making it a fast transporting compound (response time <20 ms). Redraw from Reference [44], with permission.

In polymer material, the detailed analysis of the molecular energy levels has led to a better understanding of the charge transport mechanism and space charge field formation [58]. Most notably, the comparison between the time constant of polyvinylcarbazole (PVK)-based material versus poly(acrylic tetraphenyldiaminobiphenyl) (PATPD)-based polymer showed that deep charge trapping can slow down the response time of the material [44]. In Figure 2, the energy levels of PVK and PATPD-based photorefractive materials are presented. On the left panel, it can be seen that, because the HOMO of the PVK polymer is relatively low (5.92 eV), deep traps are present in the form of chromophores 7-DCST (4-homopiperidinobenzylidenemalononitrile) and DBDC (3-(N,N-di-n-butylaniline-4-yl)-1-dicyanomethylidene-2cyclohexene) molecules with levels at 5.90 eV and 5.62 eV, respectively. These deep traps attract the charges, slowing down their transportation along the polymer manifold. The PVK-based photorefractive material has a response time measured in the hundreds of milliseconds. On the right panel, the PATPD HOMO level is too high (5.43 eV) for the holes to be trapped into the 7-DCST or DBDC molecular levels, allowing for a faster charge transport and a photorefractive effect with a response time measured in the tens of milliseconds.

Most recently, it also has been demonstrated that, by reducing the dispersion of the energy levels in the polymer manifold, a faster charge transport can be accomplished. This happens because the charge is not trapped in local maximum but is, rather, transferred from molecule to molecule without loss of momentum [59]. In addition, the role of the plasticizer as trapping site has been reported to achieve very high refresh rate and diffraction efficiency [60].

### 3.3. Molecular Species in Photorefractive Polymers

**Figure 3:**
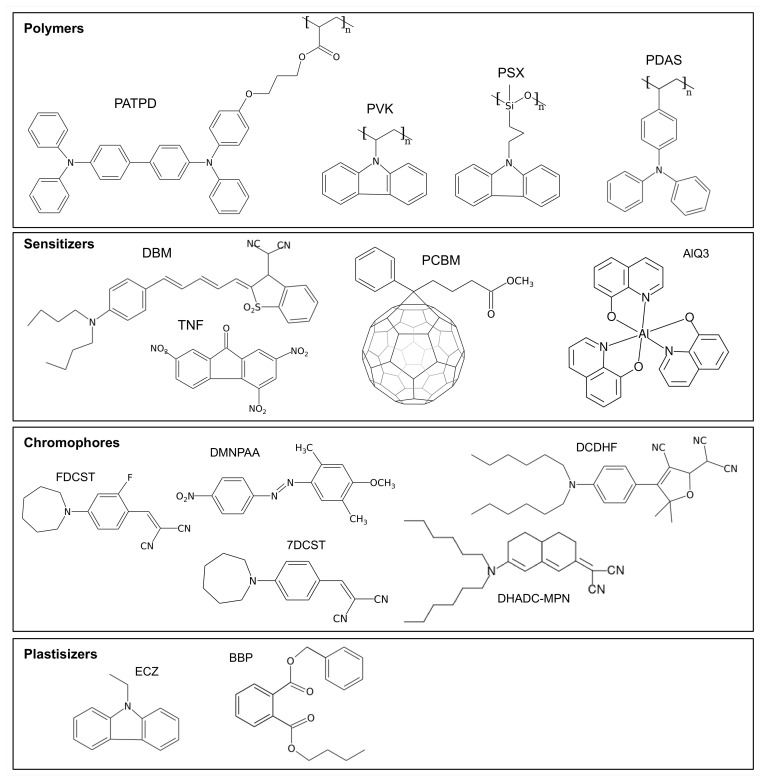
Structure of the most commonly used molecules found in photorefractive polymers. See text for chemical name and material composition.

The most widely used sensitizer molecules in photorefractive polymers are 2,4,7-trinitro-9-fluorenone (TNF) and phenyl-C61-butyric acid methyl ester (PCBM) (see Figure 3 for their chemical structure). TNF is only used in combination with the PVK polymer since, together, they form a charge transfer complex that is sensitive to the visible light. PCBM is a $C_{60}$ molecule that is functionalized to improve its solubility in the polymer matrix. Good results have also been obtained by doping the material with graphene as a sensitizer [61].

The polymer matrix used in photorefractive compounds is not only responsible for the structural integrity of the material but is also used to support charge transport. While a large array of photoconductive polymers has been used over time, the two most predominant matrices found in the literature are PVK and PATPD (see Figure 3 for their chemical structure). Both of these materials are hole conductors, meaning that the excited electrons stay in place in the excited LUMO level of the sensitizer, and it is the holes that are transported over the polymer chain by a hopping mechanism.

Once the space-charge field has been generated, the chromophores are responsible for the creation of the index modulation. Two mechanisms intervene to change of the refractive index: the electro-optic effect and the orientational birefringence. The electro-optics effect, also known as the Pockels effect, is the non-linear response of the chromophore index ellipsoid to the local electric field. This field is composed of the external applied field and the space-charge field. For a material to exhibit the Pockels effect, it cannot be centrosymmetric, or the second order hyperpolarizability (β) will be zero [62]. To break the centrosymmetry, the chromophores should be aligned, which happens thanks to the externally applied electric field.

On the other hand, the orientational birefringence, is the alignment of chromophore directly in the local electric field [63,64,65,66]. Because the chromophores are usually rod-like molecules, they possess a dielectric anisotropy (μ) and are subject to a moment that aligns them in the field. This couple is related to the first order polarizability difference between the long and short axes of the molecule (Δα). For the chromphores to be able to rotate, the polymer matrix should be pliable, which is the reason why lower glass transition temperature compounds usually exhibit better diffraction. However, the orientational birefringence is a much slower effect than the electro-optic effect and requires at least a few milliseconds to be observed [67].

To take into account both the electro-optic effect and the orientational birefringence, a new figure of merit has been devised for the chromophore used in photorefractive compound:

(1) $$FOM=\frac{1}{M}\left(\frac{2}{9k_{b}T}\Delta \alpha \mu^{2}+\beta \mu \right),$$

where *M* is the molecular mass, $k_{b}$ is the Boltzmann constant, and *T* is the temperature.

**Figure 4:**
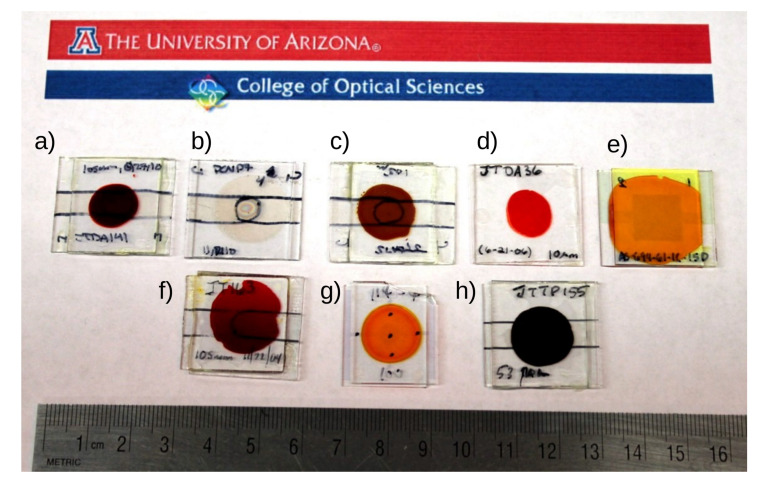
Picture of various photorefractive samples illustrating the different absorption the samples can exhibit: (**a**) PVK:DHADC-MPN [42], (**b**) PVK without chromophore, (**c**) PVK:TNF:DMNPAA [69], (**d**) PVK:DR1 [70], (**e**) PATPD:FDCST [32], (**f**) PATPD:7DCST:DBM [43], (**g**) PATPD:7DCST:AlQ3 [71], (**h**) PATPD:PCBM [53].

This figure of merit has important consequences for the coloration of the chromophores and their use in holographic 3D display. Indeed, highly conjugated molecules with a high β also exhibit a high absorption in the visible spectral range, preventing the transmission of some colors [68]. As it can be seen in Figure 4c, the sample doped with DMNPAA has a deep brown color that absorbs both green and blue. FDCST-doped material, seen in Figure 4e, is orange, letting green light go through. Although FDCST has a lower hyperpolarizability, its large birefringence is compensating for it, so that this molecule has a large FOM.

More recent works have shown that very high sensitivity can be obtained with poly(triarylamine)s (PTAA) compounds, thanks to their high dipole moment that allows for a large concentration of chromophores to be loaded in the polymer matrix [72,73]. Another approach calls for reducing the amount of space the chromophore needs to align in the field. By controlling the chromophore free volume, researchers have demonstrated a faster photorefractive response [74,75].

To improve the efficiency and shorten the response time of photorefractive polymers, plasticizer molecules, such as BBP (benzyl butyl phtalate) or ECZ (ethyl carbazole), are often added into the compound (see Figure 3 for their chemical structure). The plasticizer molecules lowers the glass transition temperature ($T_{g}$), which improves the mobility of the chromophores [41,63,76]. However, a side effect of lowering the $T_{g}$ is to make the material susceptible to crystallization over time. The transformation from amorphous to crystallized manifests itself in polymer by making the material opaque, which is obviously a problem for optical applications. Fortunately, the crystallization mechanism is fully reversible, and the transparency of the sample can be restored by heating it above the $T_{g}$ for a few seconds. The sample then needs to be quenched to room temperature to avoid immediate re-crystallization.

By reheating the material at regular intervals, from weeks to years, depending of the composition, the lifetime of the sample can extended indefinitely. The sample used in our 3D display setup presented in 2008 is still functional to this day, 13 years later [77].

From our observation over the years, the photorefractive effect in polymer is fully reversible and does not present any fatigue. Bleaching of the chromophore can happen if high energy density recording beams are used to write the hologram. Otherwise, the only sample failure is the catastrophic electrical breakdown that happens between the electrode when the high voltage is applied (see next section for details on the external field). This electrical breakdown releases the potential energy in the form of a high voltage current that chars the molecules and forms a conductive channel. Once that channel has been created, the sample cannot hold any voltage and does not exhibit diffraction any longer. The dielectric breakdown is often initiated at the location of micro bubble, lint, or other defect in the sample. To reach high applied voltage (>6 kV) without breakdown, the sample needs to have a high purity and uniformity.

## 4. External Field and Sample Geometry

The charge transport is assisted by the application of an external electric field. That same field is also responsible for helping the dissociation of the charge bound pair in the sensitizer. However, in the case of the charge transport, the external field orientation ($\vec{E}$) relative to the grating vector ($\vec{K}$) is important for the generation of the space-charge field.

**Figure 5:**
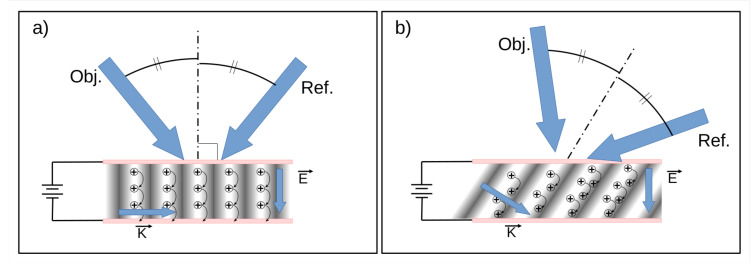
Geometry of the external field ($\vec{E}$) with respect to the grating vector ($\vec{K}$). (**a**) When the grating vector is orthogonal to the external electric field, the charges generated in the bright regions are driven towards the opposite electrode where they are collected. No space-charge field is created, and no diffraction is observed. (**b**) By tilting the grating vector, the charges are pulled toward the dark regions of the grating where they can be trapped, forming the space-charge field.

The grating vector direction is defined as being parallel to the modulation of the interference grating. When the external electric field vector is orthogonal to the grating vector, the charges that have been generated in the bright regions of the interference grating are transported along these bright regions and collected by the opposite electrode. In doing so, the charges are never driven toward the dark fringes, and no space-charge field can be established (see Figure 5a).

In order to pull the charges in the direction of the dark regions of the grating, the external field should have a component along the grating vector. This is done by tilting the sample according to the bisector of the reading beams angle, to obtain a non-symmetrical geometry. This geometry, represented in Figure 5b, has important consequences for the development of a color 3D display, as we will see later.

Recently, some compounds have been reported to show photorefractive behavior without the needs to an applied electric field [78,79,80]. This is an interesting development that especially helps with the samples’ manufacturing, as well as their long term use. However, one should also consider that, without an external field, the material sensitivity and efficiency is lower, and a field might still be required for practical utilization.

## 5. Photorefractive-Based 3D Display

### 5.1. Early Works and Rational

As presented in the introduction, the first example of holographic animation was demonstrated using motion picture holography [15,16]. As in regular 2D motion pictures, the holograms were recorded on permanent media, such as silver halide film, and then projected at a fast enough succession rate that generates the perception of movement. However, when dealing with permanently recorded holograms, holographic motion pictures cannot be updated in real time. For that, the display needs a dynamic holographic material.

Early attempts at making a refreshable holographic display included the use of photorefractive crystals, such as strontium barium niobate (SBN:60) [81,82]. The problem at the time was the small size of the crystals (about 20mm×20mm), which limited the extent of the image that could be displayed. It has to be noted that there are still some efforts in that direction by growing larger crystals (up to 60mm×40mm) of bismuth and magnesium co-doped lithium niobate [83].

**Figure 6:**
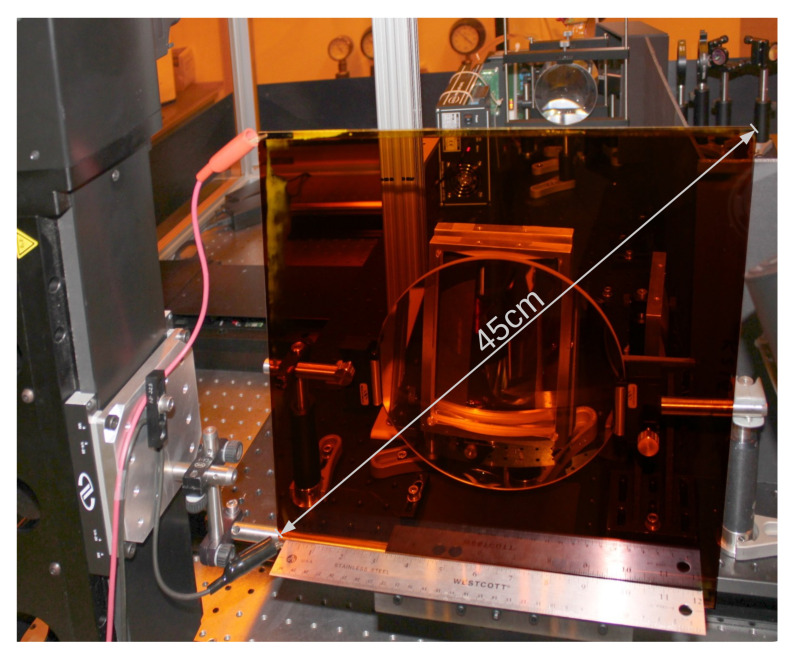
Picture of a large 45-cm diagonal photorefractive screen in front of the optical setup used to record the holograms. The sample is composed of a 100μm layer of photorefractive polymer sandwiched between two glass plates; the inner sides of the glass plate are coated with indium tin oxide (ITO) transparent electrode. The red and black cables attached to the sample are providing the voltage for the external electric field.

The benefit of a photorefractive polymer material is that it can be made into large films without too many difficulties. To date, the largest photorefractive screen is 450 mm in diagonal and can display a 3D image about the same size (see Figure 6) [32].

The other advantage of the photorefractive polymers that makes them highly suited for 3D display is their relatively high sensitivity. Sensitivity is one of the most important characteristics of any holographic recording material, dynamic or permanent, and is defined as:

(2) $$S=\frac{\sqrt{\eta }}{I_{tot}Lt},$$

which relates the diffraction efficiency (η) to the total writing energy ($I_{tot}$), the material thickness (*L*), and the exposure time (*t*).

The unique feature of photorefractive materials over any other dynamic holographic recording mechanism, such as molecular hole burning, molecular reorientation, or photobleaching, is that the sensitivity of photorefractive media can be improved by increasing the external applied voltage. The voltage and, thus, the efficiency is only limited by the dielectric breakdown value ($E_{max}$) at which the material experiences a catastrophic failure.

It has been demonstrated that adding a dielectric buffer layer between the electrode and the polymer can increase the value of $E_{max}$ [84]. The maximum field value can reach up to 100 V/μm and is leveraged to improve the efficiency to around a tenth of (mJ/cm${}^{2}$· 100 μm)${}^{-1}$ (e.g., 100% efficiency can be reached with $10\;\mathrm{mJ}/{\mathrm{cm}}^{2}$ of optical power in a 100 μm thick film). This makes photorefractive polymers among the most sensitive refreshable holographic recording materials.

Table 1 presents some of the photorefractive polymers used in holographic displays, along with their important metric to consider when recording holograms. The materials are organized by decreasing recording time.

### 5.2. Holographic Stereograms

Having a large, sensitive, refreshable holographic material is only one part of the task for making a 3D display. The other part is being able to feed that material with the information needed to display the hologram.

The dynamic capability of photorefractive materials is often demonstrated by recording the changing images displayed on a spatial light modulator (SLM) [60,87]. However, the image coming from these SLMs is only 2D. Therefore, the hologram recorded in the holographic material using SLMs is also 2D and lacks depth information. To display 3D images, another approach needs to be taken, one of which is holographic stereograms.

Holographic stereograms have been developed to reproduce depth information, such as parallax and occlusion in holographic materials [88,89]. In a holographic stereogram, the entire hologram is recorded pixel by pixel, filling the plane of the material. These pixels are called hogels and are, in fact, holograms [90].

**Figure 7:**
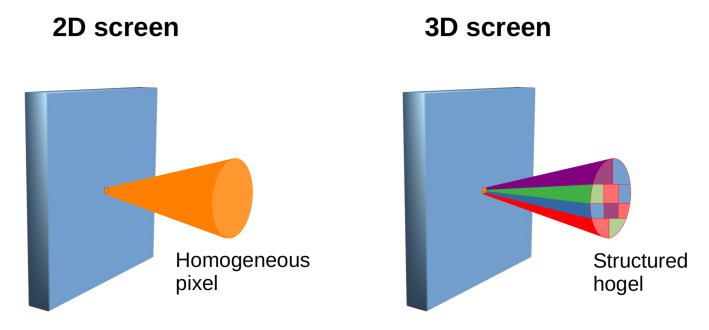
Difference between a pixel from a 2D screen that emits a homogeneous cone of light, and hogel from a 3D stereogram that emits a structured cone of light.

As presented in Figure 7, regular pixels found in any 2D display devices emit a cone of light that is the same in all directions. On the other hand, a hogel diffracts a cone of light that has an internal structure. The hogel cone of light changes intensity and color according to its direction. As such, the viewer sees different colors emitted by the hogel according to is location.

The collection of hogels recorded in a holographic stereogram display different images in different directions. These different images can be such that they reproduce 3D cues (parallax and occlusion). These images can also be organized in such way that they reproduce movement when the viewer move in front of the display. In the case of holographic data storage, the different angles contain totally different information for angular multiplexing [91,92].

Even used with permanent recording material, holographic stereogram printing technique is the subject of intense research to improve resolution, depth of field, field of view, color gamut, and other aspects of the 3D image [93,94,95].

### 5.3. Display Engineering: Ever Faster

For permanent image recording, the printing speed is not too much of a concern. However, for dynamic display application, the recording of the holographic stereogram should be fast enough so that it refreshes the image at short intervals. There are several key elements of the printing system that define the printing speed. First are the photorefractive material response time and sensitivity. The response time should be fast enough so that only a short exposure time is required to record a hogel. Likewise, the material should be sensitive enough so that only a small amount of light is required to obtain a high diffraction efficiency. However, it has been shown that photorefractive materials have a reduced sensitivity when exposed with nanosecond pulses, and more energy is required to achieve the same efficiency than with longer exposure time [84].

Nanosecond pulse exposure has the advantage of not being sensitive to any vibration, which makes the holographic system much more simple. In addition, fast repetition rate (100 Hz) lasers, with hundreds of millijoules of power per pulse are readily available in that range of pulse width. Such a large power per pulse is needed because of the material’s relatively low-sensitivity (in the order of mJ/cm${}^{2}$).

Another element to consider for fast recording speed is the image device that modulates the object beam intensity profile. The leading technology is the liquid crystal on silicon (LCoS) SLM. However, these SLMs are usually limited to a refresh speed of 180 Hz (3 color at 60 Hz). For an even faster refreshing rate, a contender is the Texas Instruments DLP, that has a refreshing rate of up to 20 kHz. Texas Instruments is also developing a micro-electro mechanical system (MEMS) capable of modulating the phase of the laser beam [96,97]. Such a device, used for holographic printing, would have the advantages of being able to make the image, focus the beam, and scan the material, all at the same time.

Lastly, the scanning system that moves the recording beams from one hogel to the next should be fast and stable. Considering that the size of the hogel defines the lateral resolution of the image, it should not be larger than 0.5 mm. For a screen that is 30 cm, 600 hogels need to be recorded for horizontal parallax only (360,000 for full parallax). Unfortunately, it is not possible to use a galvanometric mirror to raster scan the screen and print the hogels. That is because the object beam needs to be tightly focused on the screen to produce a cone of light with a large angular aperture. This is made possible by using a lens that moves right in front of the screen. Early setups used a translation stage to move that lens [32,77]. However, the speed and mass of the stage limits the refreshing rate to about two holograms per second. It also requires the stage to move back and forth to complete the scan of the entire surface of the material which is that source of vibration.

A solution that has been proposed is to use flexible holographic lenses mounted on a belt that circulates in front of the screen. Because the lenses are flexible, they can be turned around in a loop, rather than going back and forth, such as a translation stage has to do. Such a system has demonstrated the fast (1 Hz) and continuous recording of holographic images [98]. The system was only limited by the repetition rate of the laser (100Hz=100 hogels/second). However, the flexible holographic lenses by themselves can easily be translated at high speed in front of the material to write the hologram at 30 Hz (30×600 hogels/second).

### 5.4. Full Color Holograms: Angular and Polarization Multiplexing

When using a photorefractive polymer for holographic recording, there are some specific considerations to take into account to optimize the diffraction efficiency, namely beam polarization, and sample orientation. Because the chromophore molecules are mostly oriented along the external electric field, the index modulation experienced with a p-polarized reading beam is larger than with an s-polarized beam. For large angles between the recording beams, these beams should be s-polarized to maximize the fringe contrast. When the reading beam ($\lambda_{R}$) has a different wavelength than the writing beams ($\lambda_{W}$), the incidence angle ($\theta_{R}$) should be corrected using the Bragg formula:

(3) $$sin\left(\theta_{R}-\psi \right)=\frac{\lambda_{R}}{\lambda_{W}}sin\left(\theta_{W1}-\psi \right),$$

where angles are measured inside the material according to the normal to the sample, and ψ is the bisector between the writing beam angles $\theta_{W1}$ and $\theta_{W2}$.

When changing the polarization between writing and reading beams, it is also important to recognize that, because photorefractive materials can be highly birefringent, the indices of refraction experienced by the s- and p-polarized beams are different. This affects the external angles of incidence very much, and a correction must be applied to maximize the efficiency.

**Figure 8:**
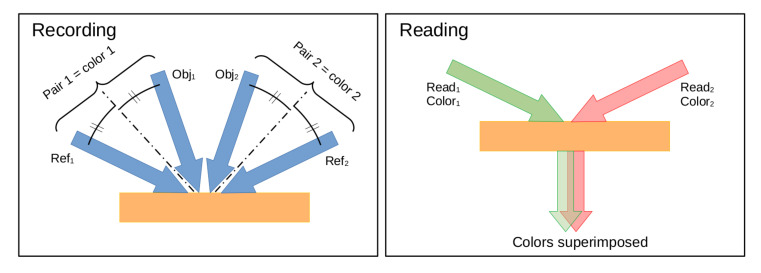
Schematic diagram for recording (**left**) and displaying (**right**) of a 2-color hologram with a photorefractive polymer.

We already discussed in Figure 5 that, to generate a space-charge field in photorefractive polymer, the geometry of the writing beams should not be symmetrical, and that a tilt angle should be applied. This can actually be used to our advantage to record a colored hologram with multiple pairs of beams, while preventing them from interfering with one another. In this multiplexing scheme, presented in Figure 8, the recording is done simultaneously with a single recording laser for both pairs of beams. The first color is recorded with a pair of beams tilted in one direction, while the second color is recorded with a pair of beams tilted in the opposite direction. In an ordinary holographic recording material (photopolymer or dichromated gelatin), such beam configuration will lead to the recording of 6 holograms: $\left\{{\mathrm{Obj}}_{1},{\mathrm{Ref}}_{1}\right\}$, $\left\{{\mathrm{Obj}}_{2},{\mathrm{Ref}}_{2}\right\}$, $\left\{{\mathrm{Obj}}_{1},{\mathrm{Ref}}_{2}\right\}$, $\left\{{\mathrm{Ref}}_{1},{\mathrm{Obj}}_{2}\right\}$, $\left\{{\mathrm{Obj}}_{1},{\mathrm{Obj}}_{2}\right\}$, $\left\{{\mathrm{Ref}}_{1},{\mathrm{Ref}}_{2}\right\}$, with the last 4 not desirable. In a photorefractive polymer, because the directions of the recording beams are symmetrical, the interferences created between $\left\{{\mathrm{Obj}}_{1},{\mathrm{Obj}}_{2}\right\}$ and $\left\{{\mathrm{Ref}}_{1},{\mathrm{Ref}}_{2}\right\}$ do not create a space-charge field, and these holograms are not recorded in the photorefractive material. The two other non-desirable interferences, $\left\{{\mathrm{Obj}}_{1},{\mathrm{Ref}}_{2}\right\}$, $\left\{{\mathrm{Ref}}_{1},{\mathrm{Obj}}_{2}\right\}$, are also much weaker for the same reason: the tilt angle is smaller.

When recording a color hologram, the angles of the recording beams should also be calculated so that, when the hologram is read with the intended wavelength, the diffracted direction is the same for both colors. This is presented in the right panel of Figure 8, where the diffracted beams superimpose in the direction of the viewer.

To add a third color and produce a full red, green, and blue gamut, a third set of recording beams can be added without causing unwanted interference by using a polarization orthogonal to the other sets of beams. Using p-polarized recording beams slightly reduces the interference pattern contrast, but this could be compensated by using that polarization to record the color that is the most efficiently transmitted (usually red).

**Figure 9:**
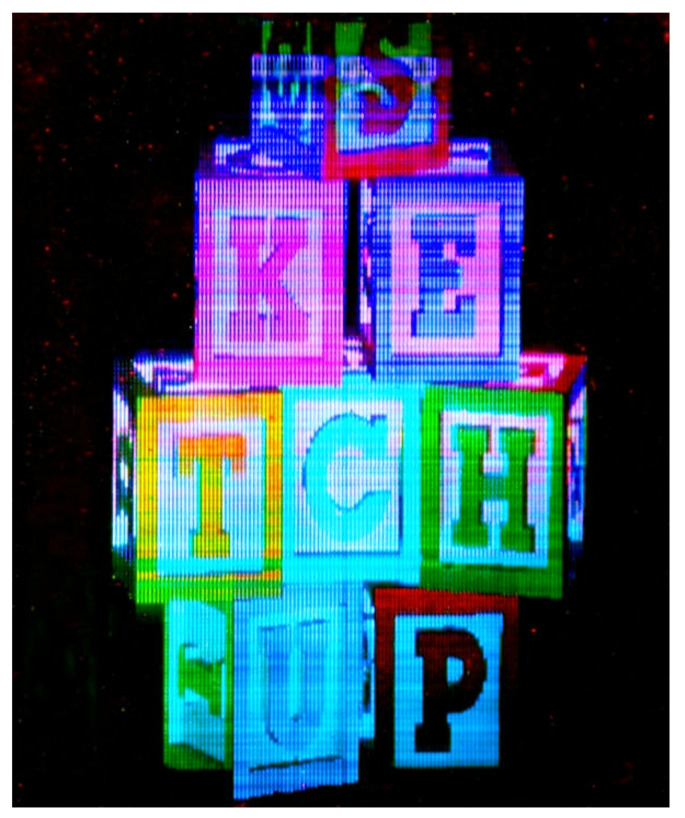
Picture of a full color (red, green, and blue) holographic stereogram recorded in photorefractive polymer by taking advantage of the symmetry and polarization of the recording beams, as described in the text. The material composition was TPD:CAAN/FDCST:BBP:PCBP (56.2:33.7:9.8:0.2).

By using all these different techniques, large, full color, quickly refreshable holographic stereograms have been recorded in photorefractive polymers. An example of such hologram is presented in Figure 9. Seen in real life, these holograms present vivid colors and striking 3D features, such as occlusions and parallax rendering.

## 6. Conclusions

The discovery, development, and optimization of photorefractive polymers is a chemical engineering success story. It took the effort of multiple research teams, and the expertise of many talented individuals to understand, characterize, and improve upon the various mechanisms responsible for the diffraction efficiency in these materials.

With the synthesis of highly sensitive compounds, the development of updateable holographic 3D display was made possible. Early demonstration presented an image of only a few ${\mathrm{cm}}^{2}$ with a refresh rate of 30 s per hologram. Now, a screen as large as $1000\;{\mathrm{cm}}^{2}$, and a refresh rate of 1 Hz, has been demonstrated, together with full color holograms.

This venture is still the subject of active research, as the recent publications made about that topic demonstrate (see Figure 1). There are still a lot of opportunities to refine the holographic image quality, reduce the optical package, and synthesize even better materials.

On the other hand, the equipment needed to record holographic 3D images on photorefractive material is relatively expensive and exotic compared to what the display industry is used to. This includes laser(s), optical lenses, and a high voltage power supply. The size of the whole apparatus is quite cumbersome, too, especially compared to flat screen television sets. This certainly needs to be improved. If the sensitivity of photorefractive materials could be reduced by yet another factor of ten to achieve high efficiency with only micro-joule of illumination per ${\mathrm{cm}}^{2}$, it would be possible to use compact pulsed fiber lasers to record the holograms. Such laser source would allow for further increase of the refreshing rate, as well as miniaturization of the optical recording system.

This would make photorefractive 3D display competitive with other more mature technologies and will eventually allow its emergence in the marketplace.

## Figures and Tables

**Table 1:** Recording metrics for some of the photorefractive polymers used for holographic display application.

Name (wt%)	Recording Power (* Energy) Density	Recording Time	Applied E Field (V/μm)	Reference
PATPD/CAAN:FDCST:ECZ				
(50:30:20)	100 mW/cm${}^{2}$	2 s	90	[77]
PDAA:7-DCST:BBP:PCBM				
(55:40:4:1)	172 mW/cm${}^{2}$	0.5 s	40	[85]
TPD:CAAN/FDCST:BBP:PCBM				
(56.2:33.7:9.8:0.2)	1000 mW/cm${}^{2}$	30 ms	72	[84]
C${}_{60}$:PbS:PATPD:PVK:7-DCST:ECZ				
(31.5:35:30:0.5:3)	1275 mW/cm${}^{2}$	1 ms	10	[86]
PTAA:PDCST:TAA:PCBM:BPhen				
(31.5:35:30:0.5:3)	534 mW/cm${}^{2}$	0.5 ms	12	[59]
PATPD/CAAN:FDCST:ECZ:PCBM				
(49.5:30:20:0.5)	650 mJ/cm${}^{2}$(*)	6 ns	70	[32]

(*) Because the recording in this material is done using a nanosecond pulse instead of continuous wave (CW) beam, we noted the recording energy density in mJ/cm${}^{2}$ per pulse instead of the power density (mW/cm${}^{2}$).

## Data Availability

Not applicable.
